# Extended reality as a training method for medical first responders in mass casualty incidents: A protocol for a systematic review

**DOI:** 10.1371/journal.pone.0282698

**Published:** 2023-03-23

**Authors:** Lucía Otero-Varela, Ana María Cintora, Salvador Espinosa, María Redondo, Miriam Uzuriaga, Myriam González, Mario García, Jessica Naldrett, Juan Alonso, Tatiana Vazquez, Alberto Blanco, María del Carmen Cardós Alonso

**Affiliations:** 1 Servicio de Urgencias Médicas de la Comunidad de Madrid (SUMMA112), Madrid, Spain; 2 Fundación para la Innovación e Investigación Biosanitarias en Atención Primaria (FIIBAP), Madrid, Spain; 3 Departamento de Enfermería, Universidad Complutense de Madrid, Madrid, Spain; Xiamen University - Malaysia Campus: Xiamen University - Malaysia, MALAYSIA

## Abstract

**Introduction/Background:**

Mass-casualty incidents (MCIs) and disasters require an organised and effective response from medical first responders (MFRs). As such, novel training methods have emerged to prepare and adequately train MFRs for these challenging situations. Particular focus should be placed on extended reality (XR), which encompasses virtual, augmented and mixed reality (VR, AR, and MR, respectively), and allows participants to develop high-quality skills in realistic and immersive environments. Given the rapid evolution of high-fidelity simulation technology and its advantages, XR simulation has become a promising tool for emergency medicine. Accordingly, this systematic review aims to: 1) evaluate the effectiveness of XR training methods and 2) explore the experience of MFRs undergoing such training.

**Methods:**

A comprehensive search strategy will encompass four distinct themes: MFRs, disasters/MCIs, education and simulation, and XR. Four databases (MEDLINE, EMBASE, CINAHL and LILACs) will be searched along with an in-depth examination of the grey literature and reference lists of relevant articles. MetaQAT will be used as a study quality assessment tool and integrated into Covidence as part of the data extraction form. Given the predicted high heterogeneity between studies, it may not be possible to standardise data for quantitative comparison and meta-analysis. Thus, data will be synthesised in a narrative, semi-quantitative manner.

**Discussion:**

This review will examine the existing literature on the effectiveness of XR simulation as a tool to train MFRs for MCIs, which could ultimately improve preparedness and response to disasters.

**Trial registration:**

**Protocol registration:** PROSPERO CRD42021275692.

## Introduction

Mass casualty incidents (MCIs) and disasters are unforeseeable events that require an organised and effective first-line response from emergency medical service providers, also referred to as medical first responders (MFRs). These highly demanding situations typically entail providing urgent medical care to a large number of victims at the same time, with the scarcity of technical resources [1, 2]. Under these circumstances, the classification of victims or triage is a dynamic and always-evolving process that depends on the demands of the environment and the severity of the patient’s injuries. Accordingly, MFRs are faced with difficult decisions to determine the preferential care and priority of evacuation for each patient, considering their chance of survival [3, 4]. As MCIs and disasters are globally increasing [5], it is paramount that MFRs are prepared and adequately trained for these challenging situations/environments/settings.

Traditional training techniques for disaster preparedness are broad and varied: from lectures and seminars, or practical tabletop exercises, to live drills including cards, actors or even sophisticated mannequins [6–8]. In recent years, simulation-based education has grown in popularity, as it offers multiple advantages, such as the possibility to train in safe environments without direct risk to participants (psychologically and physically) and the opportunity for repetitive practice to acquire a certain level of competence [9, 10]. Furthermore, the rapid evolution of high-fidelity simulation technology has led to the widespread use of novel training tools in medicine, with a particular focus on extended reality (XR), i.e., virtual, augmented and mixed reality (VR, AR, and MR, respectively). Such systems integrate immersive environments and realistic scenarios that allow participants to increase the speed of gaining knowledge and develop high-quality skills [11–13].

Enhancing the training MFRs receive is fundamental to strengthening the preparedness and response to MCIs and disasters. Previous systematic reviews have assessed the use of XR simulation training. However, these did not focus on the out-of-hospital emergency setting [14, 15]. Conversely, other reviews have identified relevant articles highlighting what could be a promising and novel tool for disaster medicine, although the effectiveness of the training was not assessed [9, 11]. Accordingly, this systematic review will examine the existing literature on relevant primary research studies to 1) evaluate the effectiveness of MCI training methods using XR simulation and 2) explore the experience of MFRs. Therefore, the research questions that this study will address are as follows:

- Primary question: What is the effectiveness of extended reality simulation as a tool to train medical first responders in mass casualty incidents?
- Secondary questions:
  ○ What tools and metrics were used to measure the effectiveness of the XR simulation training?
  ○ What is the experience of medical first responders training for mass-casualty incidents using XR simulation? What’s their perceived impact of such training?

## Materials and methods

This protocol is registered at the International Prospective Register of Systematic Reviews (PROSPERO #CRD42021275692) and adheres to the Preferred Reporting Items for Systematic Review and Meta-Analysis Protocols (PRISMA-P) [16].

### Eligibility criteria

Detailed inclusion and exclusion criteria are available in Table 1 and described below.

**Table 1:** Inclusion and exclusion criteria for abstract and full-text screening stages of the systematic review.

Criteria	Included	Excluded
***Abstract screening***		
Study Design	Experimental studies, Quasi-experimental, studies, Observational studies (cohort, case-control, cross-sectional), Conference proceedings Studies with unclear design	Letters, Editorials, Comments, Books, Reviews
Participants	Emergency physician/doctor Emergency nurse Emergency medical technicians Paramedics Residents (medicine and nursing) Students (medical, nursing, paramedic and technician)	Other type of healthcare providers, such as family physicians, general practitioners, surgeons, … In-hospital emergency physicians/doctors
Intervention	Training/education using extended reality (i.e., VR, MR, AR) simulation	No intervention (i.e.: lack of training)
Setting	Simulated scenarios of mass casualty incidents (natural disasters or human-made)	Other type of settings not related to the topic
***Full-text screening***		
Study Design	Experimental studies, Quasi-experimental studies, Observational studies (cohort, case-control, cross-sectional)	Letters, Editorials, Comments, Books, Reviews
Participants	Emergency physician/doctor Emergency nurse Emergency medical technicians Paramedics Residents (medicine and nursing) Students (medical, nursing, paramedic and technician)	Other type of healthcare providers, such as family physicians, general practitioners, surgeons, … In-hospital emergency physicians/doctors
Intervention	Training/education using extended reality (i.e., VR, MR, AR) simulation	No intervention (i.e.: lack of training)
Comparator	Simulation training not using extended reality, training not using simulation No control group (e.g., before and after studies)	-
Setting	Simulated scenarios of mass casualty incidents (natural disasters or human-made)	Other type of settings not related to the topic
Outcome	Effectiveness and/or participants’ perception	No outcome

#### Study types

To conduct a thorough systematic review, this review will not be restricted to interventional studies but will incorporate all original research reporting on XR training for medical first responders. Accordingly, experimental, quasi-experimental and observational studies will be included. Among these, health technology assessments and health economic evaluations are also expected to be captured.

#### Participants

This review will focus on medical first responders, defined as out-of-hospital healthcare professionals from the emergency medical service. Derivatives and variations of this term will also be accepted.

#### Intervention

Educational activities or training using extended reality (XR) simulation, including virtual reality (VR), mixed reality (MR) and augmented reality (AR) [17].

*Virtual reality*. VR is the best known of these technologies. It is fully immersive, making people think they are in a different environment or world from the real one. Using a head-mounted display (HMD) or headset, users can experience a computer-generated world with images and sounds. They also can manipulate objects and move around using touch controllers while connected to a console or PC.

*Augmented reality*. AR superimposes digital information on real-world elements. AR keeps the real world at the centre, but enhances it with other digital details, overlaying new layers of perception and complementing the reality or environment.

*Mixed reality*. MR brings together the natural world and digital elements. Using state-of-the-art sensing and imaging technologies, users interact and manipulate physical and virtual components and environments in mixed reality. Mixed reality allows users to see and immerse themselves in the world, even when interacting with a virtual environment using their own hands, without the need to remove their headsets. In addition, it provides the ability to have one foot (or type) in the real world and the other in an imaginary place, thus breaking down the basics between the real and the imaginary.

#### Comparator

Studies comparing the intervention group to controls [18, 19] (i.e., medical first responders trained using another simulation or training) will be included. Also, studies with no control group (e.g., before and after) will be selected.

#### Setting

Simulated scenarios of mass casualty incidents, including those caused by natural disasters or human-made.

#### Main outcomes

The outcomes of interest are the effectiveness of the XR simulation training for MFRs in mass casualty incidents, and the perception of MFRs. There is no standard definition for these outcomes; but instead, these will be determined based on the information reported in the included articles. For instance, participants’ experience could be interpreted as satisfaction, opinion, acceptability, or usability; while effectiveness can also have many definitions, including but not limited to triage accuracy, time to triage, or intervention correctness.

Said outcomes will be classified within Kirkpatrick levels (Table 2), given that it is the most effective and most used approach for evaluating training [20]. To better address the secondary questions, the metrics and tools used for effectiveness will also be captured.

**Table 2:** Kirkpatrick levels [20].

Levels	Definition
Level I—Reaction	A measure of how participants feel about the training program
Level II—Learning	An objective, quantifiable measure of how well trainees have acquired knowledge, improved skills, or changed attitudes due to training
Level III—Behaviour	A measure of how well behaviors learned in training are performed on the job (i.e., transfer of training)
Level IV—Outcome	A measure of how well training relates to final results, such as improved patient outcomes, reduced costs, enhanced quality

### Information sources and search strategy

The following bibliographic databases will be searched with no date, country, or language restrictions: MEDLINE (Medical Literature Analysis and Retrieval System Online), EMBASE (Excerpta Medica Database), CINAHL (Cumulative Index to Nursing and Allied Health Literature), and LILACS (*transl*. Latin American and Caribbean Literature in Health Sciences).

To capture additional studies, clinical trials registries and health technology assessment databases will be searched, namely: ClinicalTrials.gov, the WHO International Clinical Trials Registry Platform and Cochrane Central Register of Controlled Trials (CENTRAL), INAHTA (International Network of Agencies for Health Technology Assessment), and BRISA (*trans*. Regional Base for Health Technology Assessment Reports of the Americas). Furthermore, grey literature will be reviewed, including Google searches, the websites of relevant emergency medicine and disaster preparedness organisations, and the reference lists and bibliographies of the included studies. Field experts will also be contacted for further information about ongoing or unpublished studies.

The search strategy for online databases was developed by the research team and revised by a research librarian with expertise in systematic reviews. As a result, the search strategy includes four broad themes:

1. Medical first responders (participants)
2. Mass casualty incidents (setting)
3. Education and simulation (intervention)
4. Extended reality (intervention)

MeSH terms (when applicable), titles and abstracts, and keywords will be searched within each theme using the Boolean operator “OR”. These four searches will be combined using the Boolean operator “AND”. The proposed keywords and MeSH terms used in this systematic review are available in S1 Table and an example search strategy is provided in S2 Table.

### Study selection and data extraction

Both abstract and full-text screening phases will be done independently and in duplicate with the support of Covidence. This web-based software platform streamlines the screening and data extraction processes. Identified records will be compiled in the reference management software Endnote™, and then uploaded into Covidence. Titles and abstracts will be first scanned following the above eligibility criteria, and articles will be selected for full-text review if: 1) both reviewers agree or 2) the abstract does not provide sufficient information to decide. Conflicts between reviewers will be identified and discussed until an agreement is reached. Study authors will be contacted when necessary for further clarification if crucial information is missing from the included articles. Inter-rater agreement will be assessed using the Kappa statistic for both screening stages.

Two authors will independently extract relevant information about each included study: first author, publication year, country, study design, setting, number and type of participants, as well as details about the intervention and comparator. Results regarding the effectiveness of the training and how effectiveness was measured (i.e., tools and metrics) will be collected and classified within Kirkpatrick levels [20]. Participants’ experiences from the disaster training will also be captured. The accuracy of the data extraction process will be guaranteed by a third reviewer, who will ensure consensus is achieved. Additionally, the data extraction form will include the different items comprising the risk of the bias assessment tool.

### Risk of bias assessment

MetaQAT will be used as a risk of bias and study quality assessment tool [21], given its versatility. As stated in the eligibility criteria, all original studies (experimental, quasi-experimental and observational) can be included; therefore, it is preferable to use one tool that allows for evaluating individual studies regardless of their research design, like MetaQAT [22]. Assessing all studies with the same tool could help prevent possible biases and heterogeneity, as opposed to simultaneously using different tools that are specific to determined study designs (e.g., Cochrane risk-of-bias tool for RCTs).

MetaQAT is a validated tool that allows for rigorous appraisal, consisting of 8 items to assess each study’s relevancy, reliability, validity, and applicability [22]. As a result, the risk of bias will be assessed at the study level, and will be reported as high, low, or unclear.

Furthermore, data on funding will be extracted, in order to asses whether included studies are subject to direct financial conflicts of interest, such as studies funded by their developers.

### Strategy for data synthesis

If possible, data from included studies will be pooled for meta-analysis, and random-effect meta-analysis will be performed using the statistical software Stata®. However, there is expected heterogeneity in the following variables: type of MFR, participants’ demographic characteristics, previous experience with XR, intervention type (i.e., VR, AR, MR), length of training, setting, effectiveness outcomes and measurement, as well as participants’ perception. Consequently, if substantial heterogeneity is found, results will be presented in a narrative form with semi-quantitative analysis using descriptive statistics.

Identified studies (both included and excluded) will be summarised in a PRISMA flow diagram, and data extracted from those selected will be tabulated into study characteristics and summary findings. Results from included studies will be elaborated in detail: relevant elements will be reported and summarised for each type of XR simulation training. Further, to better address the research questions, study findings will be stratified by the effectiveness of the training and its classification within Kirkpatrick’s evaluation model [20]. The tools and metrics used to measure effectiveness will be described. Participants’ experience will also be outlined.

## Discussion

To our knowledge, this will be the first systematic review to evaluate the use of extended reality simulation to train medical first responders for mass casualty incidents.

Among the strengths of this review are the in-depth search strategy, the specific inclusion/exclusion criteria, and the validated quality appraisal tool (i.e., MetaQAT). Therefore, this protocol provides a rigorous template for future scoping or systematic reviews for effective training methods for medical first responders.

Outcomes of this study will interest medical first responders, policy-makers, emergency medical service agencies, and disaster management authorities. Our findings will also provide a direction for future researchers seeking to improve the training of medical first responders.

A survey of several European countries on how they train and prepare for disasters underlines the need for training programmes that emphasise the creation and use of safe training scenarios, set training objectives in medical aspects of disaster management, and provide a clearer understanding of the medical aspects of disaster management. Following this choice, they want the exercise to concentrate mainly on the prehospital medical care and management aspects. They prioritise medical coordination procedures, medical management at the site, medical alert procedures, assessment of immediate needs, medical resources management, medical transport protection and safety procedures [23]. In another European survey, the EU Commission gave the following recommendations: organise focused expert meetings on the abovementioned subjects. Promote joint exercises, collect and promote best practices by supporting research for evidence-based results, and promote cross-border cooperation and possibly preplanned reinforcements [24]. All in all, there is a perceived lack of adequate training with current methods and the need to advance in new technologies and types of simulation to achieve the proposed objectives for disaster training [25].

## Supporting information

S1 Table Systematic review search parameters: Keywords and mesh terms for search strategy (classified by theme). (DOCX)

S2 Table Search strategy for EMBASE database. Accessed on October 1st, 2021.
(DOCX)
