# Supplementary material for: Extended reality as a training method for medical first responders in mass casualty incidents: A protocol for a systematic review
Source: PLoS One. 2023 Mar 23;18(3):e0282698. doi: 10.1371/journal.pone.0282698 (PMC10035843; doi:10.1371/journal.pone.0282698)
Supplement: S1 Table — (DOCX) [file pone.0282698.s001.docx]

**S1 Table. Systematic review search parameters: keywords and Mesh terms for search strategy (classified by theme)**

| **PARTICIPANTS (Medical first responders)** | **SETTING**  **(Mass casualty incidents)** | **INTERVENTION (Education and simulation)** | **INTERVENTION**  **(Extended reality)** |
| --- | --- | --- | --- |
| Advanced Trauma Life Support Care  [Air Ambulances](https://www.ncbi.nlm.nih.gov/mesh/68017732)  [Ambulances](https://www.ncbi.nlm.nih.gov/mesh/68000552)  Ambulatory Care  Basic Life support  [Disaster Medicine](https://www.ncbi.nlm.nih.gov/mesh/68054597)  [Disaster Planning](https://www.ncbi.nlm.nih.gov/mesh/68004189)  Emergency health care  Emergency personnel  [Emergencies](https://www.ncbi.nlm.nih.gov/mesh/68004630)  Emergency care  emergency care providers  Emergency management  [Emergency Medical Dispatcher](https://www.ncbi.nlm.nih.gov/mesh/2016554)  Emergency Medical Personnel  [Emergency Medical Services](https://www.ncbi.nlm.nih.gov/mesh/68004632)  [Emergency Medical Technicians](https://www.ncbi.nlm.nih.gov/mesh/68004634)  [Emergency Medicine](https://www.ncbi.nlm.nih.gov/mesh/68004635)  [Emergency Responders](https://www.ncbi.nlm.nih.gov/mesh/68060731)  Emergency Treatment  First Aid  First Aid / Instructor  First responders [Tw]  [Humans](https://www.ncbi.nlm.nih.gov/mesh/68006801)  Medical emergency  Medical first responders (MRFs)  Medical responders  Out of hospital  Paramedic  Pre-hospital  Primary care  Primary health care  [Public Health / injuries](https://www.ncbi.nlm.nih.gov/mesh/68011634)  Risk Management | Catastrophe  CBRN threats  CBRNe threats  Climate change  Disaster Victims  [Disasters](https://www.ncbi.nlm.nih.gov/mesh/68003657)  Earthquakes  Emergency scenario  Emergency Shelter  Floods  Hazards  Highly demanding situations  High-risk situations  Human-made accidents  Human-made disasters  Injured people  [injuries [Subheading]](https://www.ncbi.nlm.nih.gov/mesh/68016544)  Landslides  Mass-casualty incidents  [Multiple Trauma](https://www.ncbi.nlm.nih.gov/mesh/68014947)  Natural disasters  Organized crime  Pandemic  Terrorist attacks  Uncommon and serious clinical situations  Unpredictable situations  Victims  [Wounds and Injuries](https://www.ncbi.nlm.nih.gov/mesh/68004631)  . | Applications  Apprenticeship  Avatars  Biosignals  Clinical education  [Communication](https://www.ncbi.nlm.nih.gov/mesh/68003142)  Communication Systems  [Competency-Based Education](https://www.ncbi.nlm.nih.gov/mesh/68003162)  Crew Resource Management, Healthcare.  Curricula  Curriculum  [Education](https://www.ncbi.nlm.nih.gov/mesh/68004493)  Education, Nursing / trends*  educational interventions  [Emergency Medical Service Communication Systems](https://www.ncbi.nlm.nih.gov/mesh/68004631)  Emergency Medical  Emergency Medical Tags.  Feedback training  Formation  Haptic feedback  Health Personnel / education*  High Fidelity Simulation Training  High-fidelity patient simulation manikins  High-fidelity simulator  Human manikin  [Interdisciplinary Communication](https://www.ncbi.nlm.nih.gov/mesh/68033183)  [Interpersonal Relations](https://www.ncbi.nlm.nih.gov/mesh/68007398)  [Interprofessional Education](https://www.ncbi.nlm.nih.gov/mesh/2051952)  [Learning](https://www.ncbi.nlm.nih.gov/mesh/68007858)  Learning curve  Learning process  Manikins  Medical Educat*  Medical training  [Nonverbal Communication](https://www.ncbi.nlm.nih.gov/mesh/68009633)  Online education  [Patient Simulation](https://www.ncbi.nlm.nih.gov/mesh/68016544)  Pretraining  [Protective Devices](https://www.ncbi.nlm.nih.gov/mesh/68011482)  Realistic experience  Real-world training  Simulation  Simulation manikin  [Simulation Training](https://www.ncbi.nlm.nih.gov/mesh/2009667)  Simulation-based  Simulation-based education / learning….  Skills  Student*  Teaching  Touch-enabled human manikin  Training  Triage skills | Adaptative smart scenarios  Adaptive Feedback in a Smartphone-Based Serious Game  App  Artificial intelligence  Audio-visual features  Augmented Reality  Augmented virtuality (AV)  body sensors  Close-to-reality  Common shared scenario  computer environment  [Computer Simulation](https://www.ncbi.nlm.nih.gov/mesh/68003198)  Computer-Assisted Instruction*  Cross-Platform (Development)  digital tools  e-learning  Enhanced realism  Extended reality (XR)  [Game Theory](https://www.ncbi.nlm.nih.gov/mesh/68005716)  game-based virtual reality  Gamification  Immersive virtual reality  interactive learning  interactive virtual environment  Mixed reality  Mixed Reality (MR) training  Mobile applications  Mobile augmented reality (MAR)  MR technique / technology  new generation of MR training  realistic scenarios  Scenario control  sensory experience  serious-gaming approaches  simulated 3-D environment  simulation laboratory  simulation performance videotapes  simulation-enhanced  Smart electronic devices  Smart scenario control  Smart wearables  telemedicine  User-Computer Interface  [Video Games](https://www.ncbi.nlm.nih.gov/mesh/68018910)  Virtual human interactions  Virtual patient  [Virtual Reality](https://www.ncbi.nlm.nih.gov/mesh/2023512)  Virtual scenarios  virtual worlds  VR  wearable technologies |
