# Supplementary material for: Extended reality as a training method for medical first responders in mass casualty incidents: A protocol for a systematic review
Source: PLoS One. 2023 Mar 23;18(3):e0282698. doi: 10.1371/journal.pone.0282698 (PMC10035843; doi:10.1371/journal.pone.0282698)
Supplement: S2 Table — Accessed on October 1st, 2021. (DOCX) [file pone.0282698.s002.docx]

**S2 Table.** **Search strategy for EMBASE database. Accessed on October 1st, 2021.**

| **Search number** | **Query** | **Results** |
| --- | --- | --- |
| 1 | exp ambulatory care/ or exp disaster medicine/ or exp disaster planning/ or exp disaster management/ or exp emergency medical dispatch/ or exp emergency medical dispatcher/ or exp emergency medical technicians/ or exp emergency medicine/ or exp emergency nurse practitioner/ or exp emergency nursing/ or exp emergency physician/ or exp emergency treatment/ or exp paramedical personnel/ or exp primary health care/ or exp rescue personnel/ or exp rescue work/ or exp risk management/ | 1102507 |
| 2 | (advanced trauma life support or air ambulance or air ambulances or ambulance* or ambulatory care or disaster medicine or disaster planning or disaster management or emergency care or emergency health?care or emergency health service or emergency health?care service or emergency medical dispatch or emergency medical dispatcher* or emergency medical service or emergency medical technician* or emergency medicine or emergency nurse practitioner* or emergency physician* or emergency responder* or emergency treatment or first aid or primary health?care or rescue personnel or rescue work or risk management or transportation of patients or triage).tw. | 150058 |
| 3 | (basic life support or emergency health?care or emergency health?care personnel or emergency health?care provider* or emergency doctor* or emergency first responder* or emergency management personnel or emergency medical personnel or emergency nurse* or first responder* or medical emergenc* or medical first responder* or medical responder* or out of hospital or out?of?hospital or paramedic or paramedical personnel or pre?hospital or pre?hospital emergency care or primary care).tw. | 224563 |
| 4 | 1 or 2 or 3 | 1239265 |
| 5 | exp biological accident/ or exp bomb/ or exp chemical accident/ or exp climate change/ or exp disaster/ or exp disaster victims/ or exp emergency patient/ or exp emergency shelter/ or exp multiple trauma/ or exp nuclear accident/ or exp pandemics/ or exp radiation accident/ or exp structure collapse/ or exp traffic accident/ or exp terrorism/ | 261973 |
| 6 | (aircraft accident* or avalanche* or biological accident* or biohazard release* or bioterrorism or bomb* or chemical accident or chemical hazard release or chemical terrorism or climate change or cyclonic storm* or disaster victim* or disaster* or drought* or earthquake* or emergency patient or emergency shelter or flood* or landslide* or mass?casualty incident* or mass disaster* or multiple trauma or natural disaster* or nuclear accident* or pandemic or radioactive hazard release or radiation accident or structure collapse or traffic accident* or terrorism or tidal wave* or tornado* or wildfire*).tw. | 281497 |
| 7 | (catastrophe or catastrophic accident or CBRN or CBRNe or high?risk situations or human?made disasters or injured people or terrorist attack* or victim*).tw. | 80048 |
| 8 | 5 or 6 or 7 | 481204 |
| 9 | exp education/ or exp emergency medical services education/ or exp emergency medical service communication systems/ or exp emergency medical tags/ or exp health care personnel management/ or exp human relation/ or exp interpersonal communication/ or exp learning/ or exp manikin/ or exp physiological feedback/ or exp psychological feedback/ or exp psychomotor performance/ or exp sensory feedback/ or exp simulation/ or exp student/ | 3701937 |
| 10 | (clinical clerkship or communication or competency-based education or continuing education or curriculum or education or educat* or emergency medical service communication systems or emergency medical tags or distance education or formative feedback or high?fidelity simulation or high?fidelity simulation training or in service training or interdisciplinary communication or interpersonal relations or interprofessional education or interprofessional relations or learning or learn* or learning curve or manikin* or medical education or nonverbal communication or nursing education or patient simulation or physiological feedback or preceptorship or protective devices or psychological feedback or psychomotor performance or simulation or simulat* or simulation training or student* or teaching).tw. | 2566861 |
| 11 | (apprenticeship or clinical education or curricula or educational activit* or educational intervention* or feedback training or interactive learning or learning process or mannequin or medical training or nursing training or simulation-based or training).tw. | 650979 |
| 12 | 9 or 10 or 11 | 5069192 |
| 13 | exp artificial intelligence/ or exp augmented reality/ or exp human computer interaction/ or exp human machine interface/ or exp microcomputer/ or exp mobile application/ or exp user-centered design/ or exp video game/ or exp virtual reality/ or exp wearable computer/ | 155580 |
| 14 | (ambient intelligence or artificial intelligence or audiovisual aids or augmented reality or data display or game theory or human?computer interaction or human machine interface or medical informatics applications or microcomputer or mobile applications or smartphone or smart glasses or user-centered design or user-computer interface or video games or virtual reality or wearable computer or wearable electronic devices).tw. | 64673 |
| 15 | (3D environment or 3?D environment or AI or augmented virtuality or close-to-reality or computer environment or digital tools or enhanced realism or enhanced reality or extended reality or game?based or gamification or haptic?based or haptic technology or immersive virtual reality or immersive virtual environment or mixed reality or realistic scenarios or real-world scenarios or smart electronic devices or smart wearables or virtual patient or virtual scenarios or virtual worlds or VR or wearable technologies).tw. | 64439 |
| 16 | 13 or 14 or 15 | 230762 |
| 17 | 4 and 8 and 12 and 16 | 528 |
